# Direct Electrochemical Addressing of Immobilized Alcohol Dehydrogenase for the Heterogeneous Bioelectrocatalytic Reduction of Butyraldehyde to Butanol

**DOI:** 10.1002/cctc.201402932

**Published:** 2015-02-20

**Authors:** S Schlager, H Neugebauer, M Haberbauer, G Hinterberger, N S Sariciftci

**Affiliations:** [a] Linz Institute for Organic Solar Cells, Johannes Kepler University LinzAltenbergerstraße 69, 4040 Linz (Austria); [b] PROFACTOR GmbHIm Stadtgut A2, 4407 Steyr-Gleink (Austria)

**Keywords:** alcohol dehydrogenase, biocatalysis, butanol, electrochemistry, reduction

## Abstract

Modified electrodes using immobilized alcohol dehydrogenase enzymes for the efficient electroreduction of butyraldehyde to butanol are presented as an important step for the utilization of CO_2_-reduction products. Alcohol dehydrogenase was immobilized, embedded in an alginate–silicate hybrid gel, on a carbon felt (CF) electrode. The application of this enzyme to the reduction of an aldehyde to an alcohol with the aid of the coenzyme nicotinamide adenine dinucleotide (NADH), in analogy to the final step in the natural reduction cascade of CO_2_ to alcohol, has been already reported. However, the use of such enzymatic reductions is limited because of the necessity of providing expensive NADH as a sacrificial electron and proton donor. Immobilization of such dehydrogenase enzymes on electrodes and direct pumping of electrons into the biocatalysts offers an easy and efficient way for the biochemical recycling of CO_2_ to valuable chemicals or alternative synthetic fuels. We report the direct electrochemical addressing of immobilized alcohol dehydrogenase for the reduction of butyraldehyde to butanol without consumption of NADH. The selective reduction of butyraldehyde to butanol occurs at room temperature, ambient pressure and neutral pH. Production of butanol was detected by using liquid-injection gas chromatography and was estimated to occur with Faradaic efficiencies of around 40 %.

## Introduction

Carbon dioxide emissions are rising and have reached the level of 400 ppm in the atmosphere.[1,2] This increase is expected to create global warming, which may cause severe meteorological events. To curb this development, a carbon capture and sequestration (CCS) in deep rocks is suggested for reducing the emissions. This approach is costly, makes no use of CO_2_, and has the risk of sudden eruptions of stored CO_2_. In our approach we follow the carbon capture and utilization (CCU) strategy, in which useful chemicals and alternative synthetic fuels are produced by photochemically or electrochemically recycling CO_2_, for example, by using solar or wind energy. Nowadays, many scientists consider CO_2_ as the next chemical feedstock and as an alternative to the petrochemical refinery.[3–5]

However, wind and solar energy are supply-driven energy sources and it is difficult to adjust production and consumption in an effective way. Thus, large-scale and transportable energy storage, such as the storage for synthetic fuels or valuable chemicals, is required. In particular, the conversion of CO_2_ into other chemicals by means of carboxylation or reduction is promising.[6,7] Carbohydrate products, such as methane or different acids and alcohols, are of high interest for the substitution of fossil fuels.[5] Higher alcohols, such as butanol, are particularly attractive owing to high energy density and direct applicability to existing combustion engines.

Nevertheless, chemical conversion of substrates like CO_2_, aldehydes, or other educts of such chemicals requires high-energy inputs. To lower the energy barriers of such reactions, homogenous and/or heterogeneous catalysts have to be applied. Besides approaches that use organic[8,9] and metal–organic[10–13] molecules as catalysts, there has also has been work on biocatalysis by using microorganisms or enzymes. The great advantage of biocatalysts is their high selectivity for specific products as well as their operation at ambient conditions.

In 1976, Ruschig et al. presented work on the enzymatic reduction of CO_2_ to formate by using formate dehydrogenase and nicotinamide adenine dinucleotide (NADH) as a coenzyme.[14] Investigations on the immobilization of three dehydrogenase enzymes in sol–gel matrices for the application in NADH-assisted CO_2_ reduction to methanol has been reported by Obert and Dave.[15] Immobilization of those enzymes in alginate matrices is suggested to improve reproducibility.[4] Studies combining enzymatic CO_2_ reduction for methanol production and immobilization of enzymes have also been reported by Xu et al.[16] The application of enzymes offers great potential for a highly selective and efficient production of fuels and chemicals by means of reduction reactions.

Such biological approaches have also been reported for the generation of butanol, which is, compared to methanol or ethanol, more favorable owing to higher energy densities. Tracy et al. showed the biological pathway of the reduction of CO_2_ to butanol by using Clostridia cultures. They used the Wood–Ljungdahl approach with two possible reduction pathways of CO_2_ to butanol by multistep enzyme catalysis. In both pathways the last step involves the reduction of butyraldehyde to butanol with the aid of alcohol dehydrogenase as catalyst.[17] Müller et al. and Chen have also presented pathways on the formation of butanol from butyraldehyde by using enzymes.[18,19]

However, almost all of these previous studies have concentrated on the use of NADH as the source of electrons and protons for CO_2_ reduction with dehydrogenase enzymes. In a different approach, direct pumping of electrons into biocatalysts offers an easy and efficient way for biochemical-assisted reduction processes in the CO_2_-reduction cascade, avoiding the use of expensive sacrificial coenzymes.

Approaches following the idea of direct electrochemical addressing, but using living microorganisms, have been demonstrated by Hasan et al. who used the electron transfer from an osmium redox polymer as a mediator to microorganisms, grown on an electrode.[20,21] Other groups investigated the use of microbial electrochemical cells for the conversion of CO_2_ to acetate,[22] methane,[23] and higher alcohols such as isobutanol.[24]

Using enzymes directly, Kuwabata et al. demonstrated the electrochemical conversion of CO_2_ to methanol with formate dehydrogenase and methanol dehydrogenase as homogenous catalysts and methylviologen or pyrrolequinolinequinone as electron mediators.[25] In another study, Reda et al. also showed the direct electrochemical reduction of CO_2_ using formate dehydrogenase, immobilized on a glassy carbon electrode by simple adsorption.[26] Through the use of an osmium redox polymer, application of enzymes to electrodes has been demonstrated by the groups of Yakovleva,[27] McKenzie,[28] and Rengaraj.[29]

In our investigations we concentrate on the final step of the biological reduction cascade from CO_2_ to butanol by direct electrochemical reduction of butyraldehyde, catalyzed by immobilized alcohol dehydrogenase. The enzyme was immobilized by using an alginate–silicate matrix on a carbon felt (CF) electrode. This matrix is expected to stabilize the immobilized enzymes on the electrode for longer operational lifetimes. As an additional advantage compared to homogeneous catalysis, immobilization of the enzymes facilitates the separation of product and catalyst and offers the opportunity of reusability of the catalyst.

**Scheme 1:**
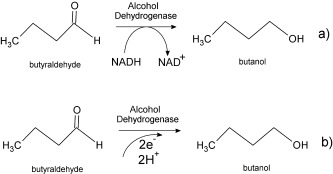
Schematic description of the reduction of butyraldehyde to butanol using a) NADH as sacrificial electron donor and b) a bioelectrocatalytic approach.

Herein, we present successful immobilization of the catalyst and efficient and selective conversion of butyraldehyde to butanol directly, without sacrificial electron-donor materials. This bioelectrocatalytic approach is presented in Scheme 1 and is compared to the conventional method with NADH.

## Results and Discussion

Initially, nonelectrochemical control experiments using alcohol dehydrogenase-containing alginate gel beads and the coenzyme NADH were performed to prove the activity of the enzymes. In this experiment samples were taken immediately after the addition of NADH and butyraldehyde, as well as after 8 h of reaction time. Analysis of the samples was performed by using liquid-injection gas chromatography.

The amount of NADH added was 7.5 mg, which corresponds to 1.127×10^−5^ moles. From gas chromatography approximately 200 ppm of butanol was determined, corresponding to a concentration of 1.067×10^−5^ moles of butanol in 4 mL of buffer solution. For each molecule of NADH one molecule of butyraldehyde is reduced to one molecule butanol, which gives an efficiency of 96 % for the production of butanol with NADH as an electron and hydrogen donor. Therefore, the results show an efficient gel bead immobilization of the enzyme.

**Figure 1:**
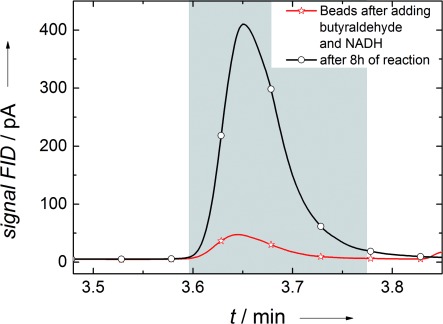
Liquid-injection gas chromatograms from a nonelectrochemical control experiment using enzyme-containing alginate gel beads and NADH as coenzyme. An intense peak is observed at the retention time of butanol (3.64 min) after 8 h of reaction time.

The chromatograms after the addition of butyraldehyde and the coenzyme NADH at the beginning of the reaction and after 8 h of reaction time are shown in Figure 1. The retention time and amount of butanol were identified by liquid-injection gas chromatography by using external and internal standards. A retention time of 3.64 min was determined for butanol. The small peak that can be observed in the chromatogram recorded right after the addition of the coenzyme and butyraldehyde indicates a fast reduction reaction to butanol with NADH as an electron and proton donor.

These results show the successful employment of alcohol dehydrogenase immobilized in alginate gel beads for the reduction of butyraldehyde to butanol with high efficiency. However, for this nonelectrochemical control experiment the expensive proton- and electron-donor NADH is required and is consumed irreversibly. Therefore, we focused on the use of bioelectrocatalysis by immobilizing the enzymes on the electrode for direct electron injection without the need for any sacrificial electron donor.

**Figure 2:**
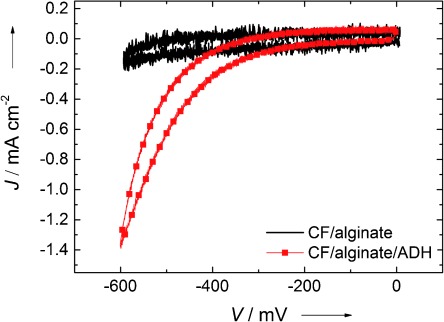
Cyclic voltammograms after the addition of butyraldehyde to the electrolyte solution, using an alginate–enzyme modified carbon felt (CF) electrode (red line) and an equally modified electrode without enzyme (black) at a scan rate of 5 mV s^−1^.

The comparison of cyclic voltammograms (CV) of an alginate-modified carbon felt electrode with immobilized alcohol dehydrogenase and an equally prepared alginate-gel-modified carbon felt electrode without enzyme, at a scan rate of 5 mV s^−1^, is shown in Figure 2. The CV’s were recorded after the addition of 0.1 mL of butyraldehyde. A reductive current can be observed, only for the electrode containing the enzyme, starting at −400 mV vs. Ag/AgCl. These results indicate that electrons can be injected directly into the immobilized alcohol dehydrogenase to catalyze the reduction of butyraldehyde to butanol.

**Figure 3:**
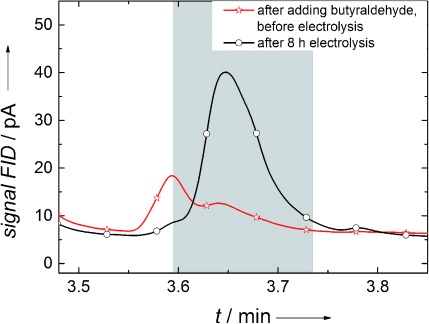
Liquid-injection gas chromatograms before and after electrolysis with butyraldehyde using a gel-modified electrode with immobilized alcohol dehydrogenase. At the retention time of 3.64 min a peak appears after electrolysis, indicating butanol generation.

**Figure 4:**
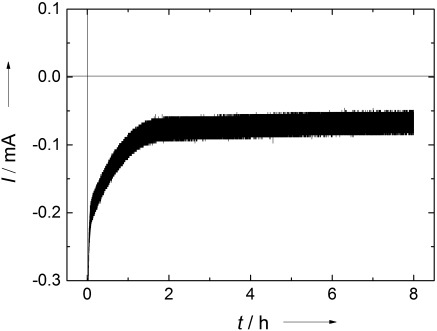
Current–time plot of the electrolysis experiment using an alcohol dehydrogenase-containing gel-modified CF electrode and electrolyte solution containing butyraldehyde.

To prove the formation of butanol by the reduction process, electrolysis experiments were performed for 8 h at −600 mV vs. Ag/AgCl. Samples of the electrolyte solution before and after electrolysis were analyzed by using liquid-injection gas chromatography. The comparison of the chromatograms before and after electrolysis is shown in Figure 3. At the retention time of butanol (3.64 min) a peak is observed according to the formation of this substance during the reduction process (the small peak at 3.59 min is related to GC-sample handling and has no significance on butanol detection). From quantitative analysis of the GC measurements an amount of 20 ppm butanol in 20 mL electrolyte solution was detected, which is calculated as 5.4×10^−6^ moles. Analyzing the current versus time curve (Figure 4), 2400 mAs were calculated from the area enclosed by the curve, which corresponds to 2.4 Coulombs (*Q*) consumed during electrolysis over 8 h at −600 mV vs. Ag/AgCl. The number of moles, *n*, can be determined by using the following equation:1

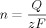


in which *F*=Faraday constant (96 485.33 C mol^−1^) and *z*=number of charges (2). The theoretical number of moles of butanol was calculated to be 1.244×10^−5^. Comparing the experimentally determined and theoretically calculated amount of butanol a corresponding Faradaic efficiency of around 40 % was found. These results support a reaction mechanism with direct electrochemical access of the dehydrogenase enzyme and subsequent reduction of butyraldehyde to butanol.

Control experiments have been performed both with enzyme-free electrodes in butyraldehyde-containing electrolyte solutions, as well as with the opposite combination, enzyme-containing electrodes in butyraldehyde-free solutions. In both cases, the chromatograms before and after electrolysis did not show any significant evidence for the generation of butanol. Butanol generation after electrolysis was only observed for electrodes containing alcohol dehydrogenase and after the addition of butyraldehyde to the electrolyte solution.

## Conclusions

The application of carbon felt electrodes modified with immobilized alcohol dehydrogenase suitable for the electrochemical reduction of butyraldehyde to butanol has been demonstrated. In control experiments, nonelectrochemical reduction by using NADH as a sacrificial electron donor has been achieved with efficiencies up to 96 %, which shows successful immobilization without the loss of enzymatic activity. To avoid the consumption of expensive coenzymes and their irreversible oxidation, an electrochemical approach for the direct enzymatically catalyzed electrochemical reduction of butyraldehyde to butanol was demonstrated. Using this direct reduction method, Faradaic efficiencies of around 40 % could be reached. Efficiencies could be improved by suppressing base currents, occurring even without the addition of enzyme or butyraldehyde. Modification of the alginate-based immobilization matrix with additional conducting materials, tuning of the electrode material, or temperature could be convenient approaches for efficiency improvement. The method of bioelectrocatalytic reduction, reported in this work, is convenient, inexpensive, and suitable for the substitution of NADH as a sacrificial electron donor. It offers the possibility of producing butanol as a high energy density chemical. Compared to homogeneous catalysis, the direct electron injection from the electrode, heterogeneously catalyzed by immobilized enzymes, facilitates the easy separation of the product and catalyst and offers the opportunity of reusability of the catalyst. The method is highly selective; butanol production is only observed for alginate-gel-modified electrodes containing alcohol dehydrogenase and when butyraldehyde is present in the electrolyte solution. Our results on the reduction of butyraldehyde to butanol with the aid of alcohol dehydrogenase show the possibility to address immobilized dehydrogenase enzymes electrochemically and suggest further use of enzyme immobilization for electrochemical CO_2_ recycling.

## Experimental Section

**Figure 5:**
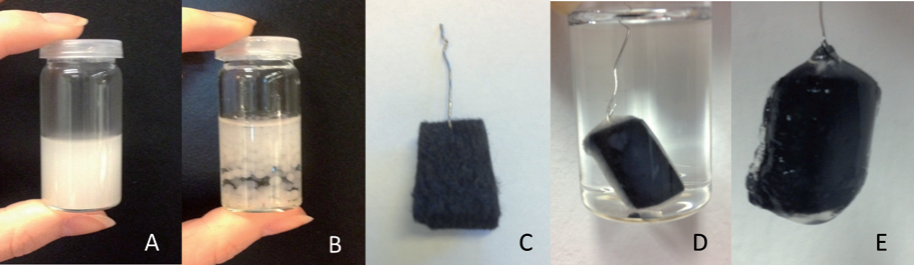
Experimental procedure: A) Alginate–silicate hybrid matrix solution containing alcohol dehydrogenase, B) gelated as beads in 0.2 m CaCl_2_ or C) soaked with a CF electrode and D) gelated in 0.2 m CaCl_2_ to obtain E) a alginate–enzyme modified carbon felt electrode.

All Chemicals were used as purchased from Sigma Aldrich. Alginic acid sodium salt was dissolved in pure water (2 mL, 18.2 MΩ) to achieve a 2 wt % solution. The solution was mixed vigorously with tetraethylorthosilicate (725 μL, TEOS). Alcohol dehydrogenase from Saccharomyces cerevisae (415 u mg^−1^ solid, 4 mg) was dissolved in aqueous 0.05 m Tris(hydroxymethyl)-aminomethane-HCl (TRIS-HCl) buffer (pH 7.65) and added to the alginate–silicate solution. For immobilization on the electrode, carbon felt (CF), purchased from SGL Carbon GmbH with a size of 0.6×3×0.6 cm, was soaked with the previously prepared matrix solution. Congelation of the alginate–silicate matrix was performed by dipping the electrode into 0.2 m aqueous CaCl_2_ solution for 20 min. The corresponding steps of the preparation procedure are depicted in Figure 5 A and C–E.

For electrochemical measurements the prepared electrode acted as the working electrode. For comparison, an alginate gel-modified electrode, but without enzyme was prepared. Pt foil was used as the counter electrode and Ag/AgCl as reference electrode. TRIS-HCl buffer (0.05 m, pH 7.65) was chosen as the electrolyte solution. Butyraldehyde (0.1 mL) was added to the electrolyte solution for the reduction reactions. All measurements were done in oxygen-free (N_2_ purged) systems. Cyclic voltammograms (CV) were recorded for both the immobilized-enzyme electrode and the electrode without enzyme, between 0 mV and −600 mV vs. Ag/AgCl with a scan rate of 5 mV s^−1^. Electrolysis was carried out for 8 h at −600 mV vs. Ag/AgCl for significant butanol production, according to the observed reduction potentials from cyclic voltammetry (Figure 2). Samples of the electrolyte solution were taken before and after electrolysis for product analysis in liquid-injection gas chromatography (Thermo Fischer, Trace 1310). All electrochemical measurements were carried out in a two-compartment cell with separated anode and cathode chamber (2×20 mL) and electrochemical measurements were recorded with a Jaissle Potentiostat-Galvanostat IMP 88 PC-R.

For comparison and enzyme-activity testing, control experiments with free-floating alginate beads, (not immobilized on an electrode), and with the aid of NADH as an electron supplier, were also conducted. For gel bead formation, the enzyme-containing gel was dropped into 0.2 m aqueous CaCl_2_ solution by a syringe, the formed beads were washed and transferred into the buffer solution (see Figure 5 A and B). The buffer solution (4 mL) containing the gel beads was purged with N_2_, followed by addition and dissolution of NADH (5 mg). Subsequently, an excess volume of the butyraldehyde (0.1 mL) was added and the reaction was conducted for 8 h. Samples were taken immediately after the addition of butyraldehyde and after 8 h of reaction time. Liquid sample analysis and product identification were performed by using liquid-injection gas chromatography (Thermo Fischer, Trace 1310).
