# Supplementary material for: Direct Electrochemical Addressing of Immobilized Alcohol Dehydrogenase for the Heterogeneous Bioelectrocatalytic Reduction of Butyraldehyde to Butanol
Source: ChemCatChem. 2015 Feb 20;7(6):967–71. doi: 10.1002/cctc.201402932 (PMC4471636; doi:10.1002/cctc.201402932)
Supplement: Supplementary file 1 [file cctc0007-0967-sd1.pdf]

Heterogeneous & Homogeneous & Bio-

CHEMCATCHEM

CATALYSIS

Supporting Information

Direct Electrochemical Addressing of Immobilized Alcohol Dehydrogenase for the Heterogeneous Bioelectrocatalytic Reduction of Butyraldehyde to Butanol

S. Schlager,*[a] H. Neugebauer,[a] M. Haberbauer,[b] G. Hinterberger,[a] and N. S. Sariciftci*[a]

cctc_201402932_sm_miscellaneous_information.pdf

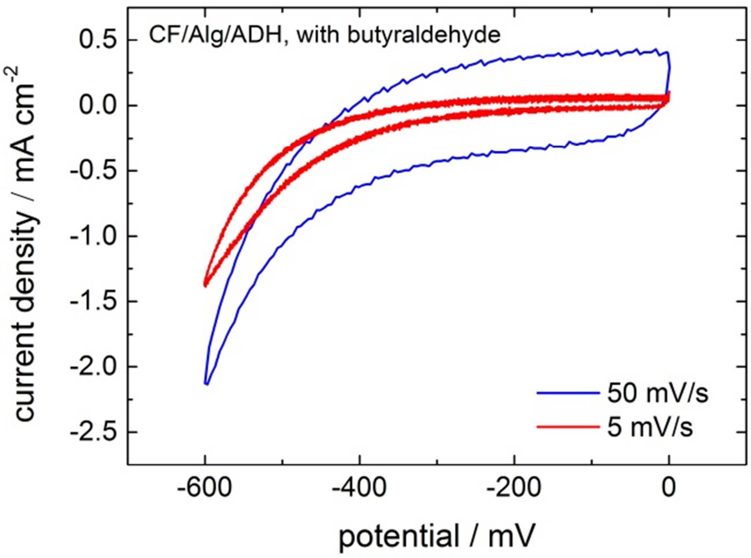

Figure 7: Cyclic voltamograms with scan rates of 50 mV/s and 5 mV/s for the reduction of butyraldehyde at an enzyme modified electrode.

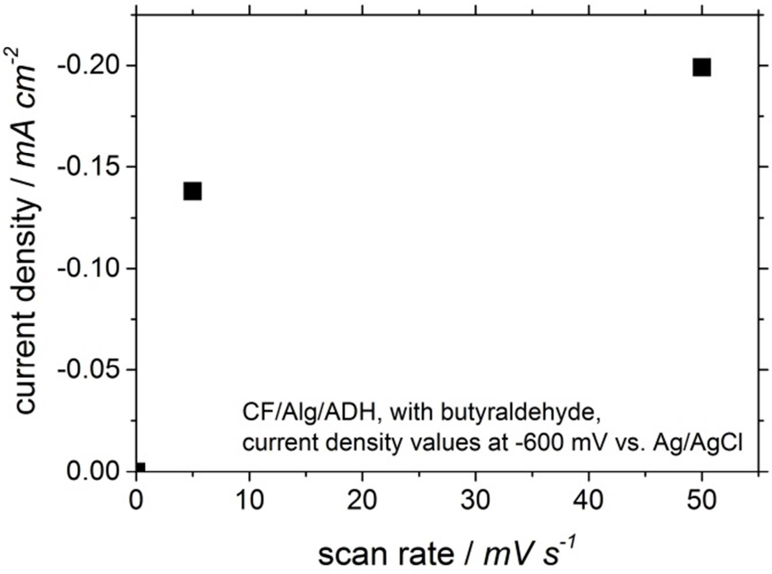

Figure 8: Correlation of current density at -600 mV and scan rate.

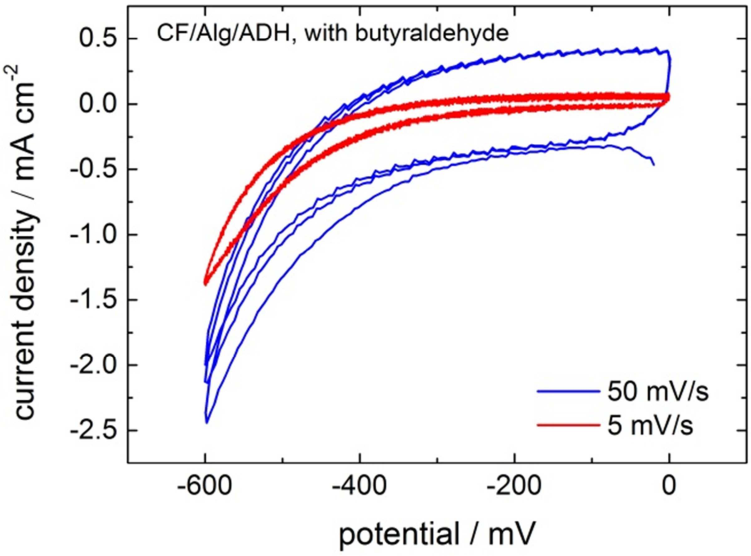

Figure 9: Cyclic voltamograms recorded with a scan rate of 50 mV/s cycling 3 times and subsequent scan at 5 mV/s.

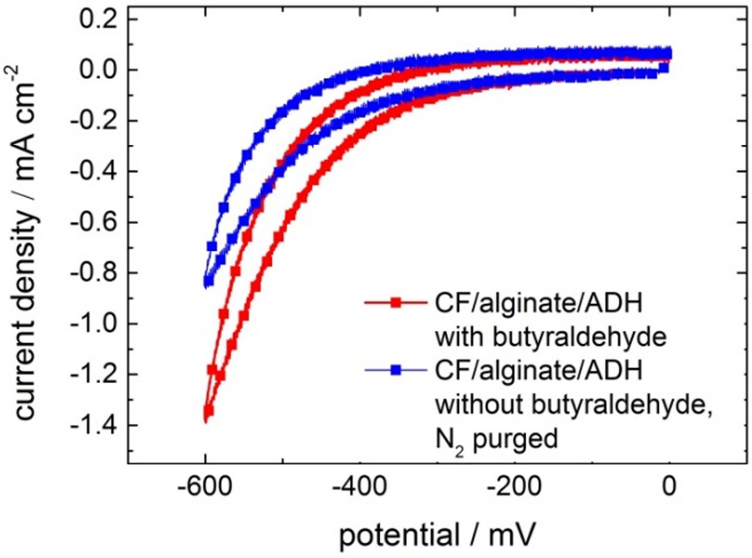

Figure 10: CF/alginate/ADH electrode in butyraldehyde containing electrolyte solution and in N_2 purged electrolyte solution without any butyraldehyde added.
